# Developmental Potential for Endomorphin Opioidmimetic Drugs

**DOI:** 10.1155/2012/715123

**Published:** 2012-06-15

**Authors:** Yoshio Okada, Yuko Tsuda, Severo Salvadori, Lawrence H. Lazarus

**Affiliations:** ^1^Faculty of Pharmaceutical Sciences, Kobe Gakuin University, Chuo-ku, Kobe 650-8586, Japan; ^2^Department of Pharmaceutical Sciences and Biotechnology Center, University of Ferrara, 44100 Ferrara, Italy; ^3^Laboratory of Toxicology and Pharmacology, National Institute of Environmental Health Sciences, Research Triangle Park, NC 27709, USA

## Abstract

Morphine, which is agonist for *μ*-opioid receptors, has been used as an anti-pain drug for millennia. The opiate antagonists, naloxone and naltrexone, derived from morphine, were employed for drug addiction and alcohol abuse. However, these exogenous agonists and antagonists exhibit numerous and unacceptable side effects. Of the endogenous opioid peptides, endomorphin(EM)-1 and endomorphin(EM)-2 with their high *μ*-receptor affinity and exceptionally high selectivity relative to *δ*- and *κ*-receptors *in vitro* and *in vivo* provided a sufficiently sequence-flexible entity in order to prepare opioid-based drugs. We took advantage of this unique feature of the endomorphins by exchanging the N-terminal residue Tyr^1^ with 2′,6′-dimethyl-l-tyrosine (Dmt) to increase their stability and the spectrum of bioactivity. We systematically altered specific residues of [Dmt^1^]EM-1 and [Dmt^1^]EM-2 to produce various analogues. Of these analogues, [*N*-allyl-Dmt^1^]EM-1 (**47**) and [*N*-allyl-Dmt^1^]EM-2 (**48**) exhibited potent and selective antagonism to *μ*-receptors: they completely inhibited naloxone- and naltrexone-induced withdrawal from following acute morphine dependency in mice and reversed the alcohol-induced changes observed in sIPSC in hippocampal slices. Overall, we developed novel and efficacious opioid drugs without deleterious side effects that were able to resist enzymatic degradation and were readily transported intact through epithelial membranes in the gastrointestinal tract and the blood-brain-barrier.

## 1. Introduction

Morphine, which represents the quintessential agonist for *μ*-opioid receptor, has been used as a pain-killing drug for millennia. Since natural occurring opioid antagonists are nonexistent, naloxone and naltrexone were derived from morphine and currently find use in drug addiction and alcohol cessation programs; however, these alkaloid-derived antagonists exhibit numerous deleterious side effects. In 1975, the endogenous opioid peptides enkephalins (H-Tyr-Gly-Gly-Phe-Met-OH/Leu-OH) were discovered [1], followed sequentially by the endorphins [2], dynorphins [3], and the endomorphins [4], all of which are involved in the modulation and attenuation of pain and regulation of homeostatic mechanisms.

 Of the endogenous opioid peptides, endomorphin-1 (EM-1: H-Tyr-Pro-Trp-Phe-NH_2_) and endomorphin-2 (EM-2: H-Tyr-Pro-Phe-Phe-NH_2_) exhibited high *μ*-opioid receptor affinity (*K_i_* = 0.36 and 0.69 nM, resp.) with high selectivity: 4,000- and 13,000-fold preference over the *δ*-opioid receptor and a similar 15,000- and 7,500-fold preference for *μ*-receptor relative to *κ*-opioid receptors [4]. These data underline the potential importance of these opioid ligands in all phases of human homeostatic mechanisms. Considering this premise, our research was directed toward the eventual development of endomorphin opioidmimetics, which would exhibit agonist and antagonist properties with potentially minimal side effects. We review the approach in this field, focusing basic research on key factors in the rational development of novel and highly efficacious opioid drugs able to resist enzymatic degradation and readily transported intact through epithelial membranes in the gastrointestinal tract and the blood-brain barrier.

## 2. Properties of Endomorphin Analogues

Opioid peptides and their G-protein-coupled receptors (*δ*, *κ*, and *μ*), which are distributed in the central nervous system and peripheral tissues, were initially classified on the basis of their functional pharmacological activity. However, despite a common mode of biological action as agonists, the structural differences among opioids permitted a division into two separate classes based on their N-terminal message domain: namely, H-Tyr-Gly-Gly-Phe-, a sequence that comprises the enkephalins, endorphins, and dynorphins, while H-Tyr-Pro-Trp/Phe- defines endomorphins-1 and -2. It is the unique sequence of the latter opioids that gave rise to their flexibility in the production of bioactive analogues.

### 2.1. Synthesis of Stereoisomeric Analogues of Endomorphin-2 and Their Activities

Initially, in order to gain insight on the interaction between opioid ligands with their receptors, we substituted d-amino acids into endomorphin-2 [5]. The rationale for the use of d-amino acids is their ability to generally affect biological activity due to a subtle change induced in peptide conformation that, if bioactive, can lead to enhanced stability against enzymatic degradation [6].

Endomorphin-2 and d-amino acid containing stereoisomers were prepared by Fmoc solid-phase method using Fmoc (9-fluorenylmethyloxycarbonyl) amide resin as follows: solid support, Fmoc-d- or l-Tyr(Bu*^t^*)-OH, Fmoc-d- or l-Pro-OH, Fmoc-d- or l-Phe-OH, and HBTU/HOBt/DMF, DIEPA/NMP were used. After each coupling reaction, the Fmoc group was removed by piperidine/NMP. For the final deblocking, dried protected peptide resin was suspended in TFA/H_2_O, and the reaction mixture was stirred at room temperature for 2 h. The material was filtered and ether added to filtrate to precipitate the peptides, which were collected by filtration and lyophilized from 1 M HCl to >98% purity. Receptor binding data are detailed in Table 1 **(2–17)** [5]. All d-amino acids containing analogues exhibited less binding affinities to the *μ*-opioid receptor (*K_i_* = 24.3–2,755 nM), resulting in the loss of high selectivity over *δ*-opioid receptor (*K_i_δ*/*K_i_μ* = 2.6–177). Interestingly, although [d-Pro^2^]EM-2 (**12**) exhibited only low affinity towards the *μ*-receptor (*K_i_* = 512.4 nM), it substantially exhibited more potent and longer activity in an *in vivo* tail flick test in mice compared to EM-2 [7]. These data clearly indicate an enhanced bioactivity most likely due to its resistance to proteolytic degradation, presumably by dipeptidyl peptidase IV [8].

### 2.2. Synthesis of [2′,6′-Dimethyl-l-tyrosine^1^ (Dmt^1^)]EM-2 Analogues: Structure-Activity Relationships

In order to develop potentially more potent analgesics, 2′,6′-dimethyl-l-tyrosine (Dmt) was substituted for Tyr as the N-terminal residue, since Dmt markedly increases the affinity and bioactivity of numerous opioid peptide agonists and antagonists [9, 14–16]. Optically pure 2′,6′-dimethyl-l-tyrosine was prepared as previously described [17].

As summarized in Tables 1 and 3, substitution of Dmt^1^ in EM-1 and EM-2 and in C-terminal deletion analogues profoundly affected all the measured parameters. In each case, the affinity of [Dmt^1^]EM-1 (**19**) and [Dmt^1^]EM-2 (**20**) towards the *μ*-opioid receptor increased 6.6 and 4.6 times compared to the parent molecules (**1**, **2**), respectively, and increased *δ*-opioid receptor affinity by 270- and 327-fold. The functional bioactivity of [Dmt^1^]EM-1 (Table 3, **19**) increased *μ*-bioactivity by 15-fold over EM-1. Interestingly, [Dmt^1^]EM-1 (**19**) was transformed to potent mixed *μ*-agonist/*δ*-antagonist, while the bioactivity of [Dmt^1^]EM-2 (**20**) greatly increased both *μ*- and *δ*-agonist bioactivities by 98- and 184-fold greater than EM-2, respectively. Similarly, the deletion of C-terminal carboxyl group of [Dmt^1^]EM-2 to yield H-Dmt-Pro-Phe-NH-C_2_H_4_-Ph (**22**) also exhibited mixed *μ*-agonist/*δ*-antagonist properties, but with over an order of magnitude less activity than those observed for **19**. The marked change in the Dmt-containing analogues relative to both receptor interaction and bioactivity could be a result of an alteration in the topography of the peptide. In fact, the ^1^H NMR spectra of EM-2 analogues revealed that the rotamers around the Dmt-Pro amide bond existed predominantly in the *cis *configuration [9].

### 2.3. Synthesis of C-Terminal-Modified [Dmt^1^]EM-2 Analogues (H-Dmt-Pro-Phe-NH-X)

**Figure 1:**
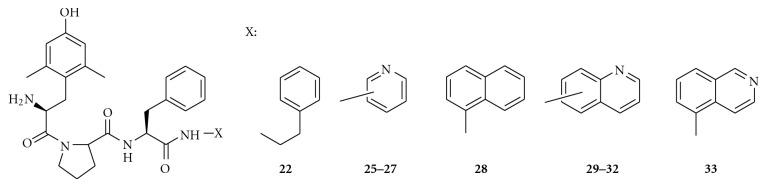
Structure of H-Dmt-Pro-Phe-NH-X.

Reports suggested that opiate tolerance and physical dependence could be blocked by *δ*-opioid receptor antagonists without compromising the antinociception produced by drug interaction at *μ*-opioid receptors [21]. From that point of view, H-Dmt-Pro-Phe-NH-C_2_H_4_-Ph (**22**) might be a candidate as an antinociceptive drug although its bioactivity was not exceptionally potent as a *μ*-agonist/*δ*-antagonist* in vitro* (GPI: IC_50_ = 5.03 nM, MVD: IC_50_ > 10,000 nM, pA_2_ = 7.05) [9]. Therefore, we substituted hydrophobic groups in lieu of the phenethyl group to develop the class of H-Dmt-Pro-Phe-NH-X ligands (Figure 1) [11]. In Tables 1 and 3, the [Dmt^1^]EM-2 analogues (**22–33**) demonstrated *μ*-opioid receptor affinity higher than EM-2 (*K_i_μ* = 0.69 nM) with *K_i_* = 0.11 to 0.52 nM, except for H-Dmt-Pro-Phe-NH-Ph (**24)** (*K_i_μ* = 1.11 nM). In terms of their *in vitro* bioactivity, they were *μ*-opioid agonists based on a guinea pig ileum (GPI) bioassay (Table 3). It should be noted that 1-Nph (**28**), 5-Qln (**30**) and 5-Isq (**33**) exhibited potent *μ*-opioid receptor agonism (IC_50 _< 1 nM). In the mouse vas deferens (MVD) bioassay, they exhibited *δ*-opioid agonism (**28**, **30**) with weak *δ*-opioid receptor antagonism (pA_2_ = 5.41–7.18). Compound **33**, a modest *μ*-agonist/*δ*-antagonist *in vitro, *produced a dose-dependent antinociceptive effect after i.c.v. administration in mice that was antagonized completely by naltrexone, indicating that its antinociception occurred through *μ*-opioid receptors similar to that of morphine [11].

These data substantiate that N-terminal Dmt-containing ligands permit development of novel bioactive opioidmimetics for potential therapeutic and clinical applications. The methyl groups on the tyramine ring of Dmt undoubtedly play a dominant role in the interaction within the opioid ligand-binding domains either by direct interaction with hydrophobic side chains of receptor residues or more interestingly by stabilization of favored *cis *conformer in solution prior to and during binding, or a combination of both mechanisms.

### 2.4. Synthesis of *μ*-Opioid Receptor Ligands Incorporating Unique Tyrosine Analogues

The enhancement of opioid activity upon inclusion of Dmt in the sequence of opioid peptides provided the impetus to develop further analogues with systematic modifications at the 2′ and 6′positions of the Tyr aromatic ring and investigate their impact on the activity of EM-2. In this study, six tyrosine analogues containing different alkyl groups were prepared, namely, 2′-monomethyltyrosine (Mmt), 2′,3′,6′-trimethyltyrosine (Tmt), 2′-ethyl-6′-methyltyrosine (Emt), 2′-isopropyl-6′-methyltyrosine (Imt), 2′,6′-diethyltyrosine (Det), and 2′,6′-diisopropyltyrosine (Dit). Opioid receptor affinities and *in vitro* functional bioactivity of the EM-2 analogues (**34–39**) are summarized in Tables 1 and 3, respectively [12]. Except for [Dit^1^]EM-2 (**38**:* K_i_μ* = 2.29 nM), the [Xaa^1^]EM-2 analogues exhibited similar or higher *μ*-receptor affinity (*K_i_μ* = 0.063–0.13 nM) to [Dmt^1^]EM-2. Evaluation of their *in vitro* bioactivities indicated that [Mmt^1^]-(**34**), [Emt^1^]- (**35**), [Det^1^]-(**37**), and [Tmt^1^]EM-2 (**39**) exhibited high GPI potencies (IC_50_ = 0.623–2.31 nM), although less than that of [Dmt^1^]EM-2 (**20**), while [Imt^1^]-(**36**) and [Dit^1^]EM-2 (**38**), which have bulky isopropyl groups, exhibited weak GPI potencies (IC_50_ = 10.6 and 299 nM, resp.). [Dit^1^]EM-2 (**38**) had unexpectedly low GPI and MVD potencies, although it retained high affinity toward both *μ*- and *δ*-opioid receptors, implying that this analogue may interact with the receptors but fail to trigger a bioactive response.

The *in vivo* biological activities of [Dmt^1^]EM-2 (**20**) and [Det^1^]EM-2 (**37**) were assessed by the induction of analgesia via the tail-flick test (spinally mediated mechanism) and hot-plate test (supraspinal effect) in comparison to both EM-2 (**2**) and morphine. The results revealed the following potency profile: [Dmt^1^]EM-2 (**20**) > [Det^1^]EM-2 (**37**) > EM-2 (**2**), which yielded activity ratios of 1.00 : 0.86 : 0.65 in the tail-lick tests and 1.00 : 0.47 : 0.30 in the hot-plate tests. These results indicated that the methyl side chain located at the 2′ and 6′ positions of Tyr represent the optimal alkyl groups for interaction with and activation of *μ*- and *δ*-opioid receptors. Interestingly, [Dmt^1^]EM-2 (**20)** was approximately 16% as effective as morphine.

### 2.5. [Dmt^1^]EM-2 Analogues Substituted at Position 3 with Alkylated Phe: Mixed *μ*-Agonist/*δ*-Antagonist and Dual *μ*-Agonist/*δ*-Agonist Opioid Ligands

The aromatic amino acid residue in position 3 is the defining structural determinant between EM-1 (Trp^3^) and EM-2 (Phe^3^). As shown, [Dmt^1^]EM-1 is a *μ*-agonist/*δ*-antagonist and [Dmt^1^]EM-2 is a *μ*-agonist/*δ*-agonist, further suggesting that the difference in chemical nature and the physical structure between Trp and Phe affected the properties of these opioid receptor ligands. Another alkylated Phe analogue, 2′,6′-dimethylphenylalanine (Dmp), was found to be an effective surrogate for phenylalanine in several opioid peptides [22, 23]. Interestingly, its replacement for Tyr^1^ in endomorphin [23] indicated that it was nearly as effective as the parental peptide, despite the absence of the important hydroxyl group on the tyramine ring, suggesting that alkylation of the aromatic ring enhances hydrophobicity and stability and/or limits rotational freedom. Therefore, we embarked on the synthesis of Phe analogues: 2′-methyl (Mmp), 3′5′-dimethyl (^3,5^Dmp), 2′,6′-dimethyl (Dmp), 2′,4′,6′-trimethyl (Tmp), 2′-ethyl-6′-methyl (Emp), and 2′-isopropyl-6′-methyl-phenylalanine (Imp) as reported [24] and their incorporation into H-Dmt-Pro-Xaa-Phe-NH_2_ [13].

As summarized in Table 1, the alkylated Phe^3^ analogues essentially enhanced the affinities for both *μ*- and *δ*-opioid receptors in these [Dmt^1^,Xaa^3^]EM-2 ligands (**40**–**46**). Of these analogues, the highest *μ*-opioid selectivity occurred with [Dmt^1,3^]EM-2 (**43**) (*K_i_δ*/*K_i_μ* = 878). One analogue of considerable interest is [Dmt^1^,Tmp^3^]EM-2 (**44**) with a 44-fold enhancement toward *δ*-opioid receptors relative to [Dmt^1,3^]EM-2 (**43**). This suggested that the hydrogen donor capacity of the hydroxyl group of Dmt was apparently less effective in affecting receptor interaction when substituted within the sequence of the peptide than the hydrophobicity of a 4′ methyl group; that is, the hydroxyl group may contribute a negative influence when it occurred as an internal residue. *κ*-Opioid receptor affinities for Dmt derivatives (**40–46**) were quite weak relative to the interaction of these peptides to both *μ*- and *δ*-opioid receptors [13].

The functional bioactivities of [Dmt^1^,Xaa^3^]EM-2 analogues generally remained essentially unchanged (**40**–**46**) relative to [Dmt^1^]EM-2 (**20**) (Table 3). Interestingly, the absence of a 4′ OH group (**42**) or its replacement by a methyl group yielded [Dmt^1^,Tmt^3^]EM-2 (**44)** and produced excellent ligands with mixed *μ*-agonist/*δ*-antagonist properties: *δ*-antagonism was 2 orders of magnitude greater than that obtained for Dmt^3^ (**43**). We have seen (*supra vide*) that [Dmt^1^]EM-1 (**19**) is a mixed *μ*-opioid agonist/*δ*-antagonist (GPI IC_50_ = 0.27 nM; MVD pA_2_ = 8.6), but [Dmt^1^,Tmp^3^]EM-2 (**44**) is obviously more potent (Table 3) [13].

These data permitted us to conclude the following: (i) the bulky side chain of Trp in combination with Dmt^1^ caused either a steric hindrance in the conformation of the peptide or a shift in hydrophobicity to potentiate the induction of *δ*-opioid antagonism; (ii) [Dmt^1^,Emp^3^]EM-2 (**45**) and [Dmt^1^,Imp^3^]EM-2 (**46**) exhibited dual *μ*/*δ*-agonism similar to that seen for [Dmt^1^]EM-2 (**20**), while compounds **40**–**44** had *δ*-opioid antagonism ranging from a weak pA_2_ = 6.59 to a potent pA_2_ = 9.05. Thus, these bifunctional molecules are targets in the design of new antinociceptive opioids that could potentially alleviate acute or chronic pain with a low degree of physical dependence and tolerance [25].

### 2.6. Transformation of [Dmt^1^]EM-1 and [Dmt^1^]EM-2 into Potent and *μ*-Selective Antagonists

The development of potent and selective opioid antagonists, especially *μ*-opioid receptor antagonists, is very important in order to delineate critical biochemical, pharmacological, and physiological roles played by these receptors and for their possible application as clinically and therapeutically relevant agents. Table 1 revealed that [*N*-allyl-Dmt^1^]EM-1 (**47**) exhibited better affinity compared to [*N-*allyl-Dmt^1^]EM-2 (**48**); however, in terms of their *in vitro* functional bioactivity (Table 3), [*N*-allyl-Dmt^1^]EM-2 (**48**) exhibited somewhat better *μ*-opioid antagonism with pA_2_ = 8.59 versus pA_2_ = 8.18 for [*N*-allyl-Dmt^1^]EM-1 (**47**) [10]. Furthermore, both antagonists are defined as neutral *μ*-antagonists due to their lack of inverse agonist properties determined by functional guanosine 5′-*O*-(3-[^35^S]thiotriphosphate) assays *in vitro* from membranes of cells grown in the presence of morphine or alcohol [26]. They also completely inhibited naloxone- and naltrexone-elicited withdrawal symptoms following acute morphine dependency in mice [26]. [*N*-Allyl-Dmt^1^]EM-2 (**48**) induced a dose-dependent suppression of an ethanol-induced increase of sIPSC frequency with full reversal at 300 nM that was several orders of magnitude more potent than naltrexone [27]. These results suggest a potential therapeutic application in the treatment of drug addiction and alcohol abuse without the adverse effects observed with inverse agonist alkaloid-derived compounds, such as naltrexone and naloxone that produce severe withdrawal symptoms.

## 3. Opioidmimetics

### 3.1. Agonists

The presence of Dmt in lieu of Tyr^1^ in opioid peptides enhanced affinities, bioactivity, and analgesia. In order to assess the possible effect of Dmt *per se* on opioid activities, H-Dmt-NH-CH_3_ was prepared and examined [28]. This compound had *K_i_μ* = 7.45 nM and *K_i_δ* = 460 nM values that were nearly equivalent to those of morphine. However, the *in vitro* bioactivity in a GPI assay was three orders of magnitude lower than that of EM-2 and [Dmt^1^]EM-2 and essentially inactive in the MVD assay. Its analgesic response relative to morphine was insignificant (0.64% in hot-plate test and 1.3% in tail-flick test). According to the message-address concept of opioid functionality [29], Dmt would be considered an important pharmacophore interacting within opioid receptors as an integral component of the message domain even though it had no intrinsic activity of its own. Thus, to test this hypothesis, we set out to construct ligands containing two message and address domains.

#### 3.1.1. Development of Receptor Agonists by Dimerization of Dmt with Unbranched Alkyl Chains

The receptor affinities and *in vitro* bioactivities of the synthetic Dmt dimer analogues are summarized in Tables 2 and 4 [18]. The *bis*-Dmt-containing ligands **52 **and **53** exhibited high *μ*-opioid receptor affinity (*K_i_* = 0.04–0.05 nM) but modest receptor selectivity (*δ*/*μ* = 1302 and 870). The optimal distance between the Dmt residues for maximum *μ*-opioid receptor affinity appeared to be butyl (**52**) = hexyl (**53**) > octyl (**54**) > ethyl (**51**). Despite the relatively good *μ*-receptor agonism of **52** and **53** (IC_50_ = 5.3 and 3.1 nM, resp.), they had undetectable *δ*-agonism and very weak *δ*-antagonism (pA_2_ = 5.5–6.4). In terms of their* in vivo *bioactivity, **52 **rapidly produced central mediated (i.c.v.) analgesia that was 1.5–2.2- fold greater than morphine and naloxone-reversible; the supraspinal nociceptive pathway revealed equivalent analgesia to morphine. Subcutaneous injection of **52 **produced analgesia that was 10–20% as potent as morphine, indicating that **52** indeed crossed epithelial membranes and the blood-brain barrier [30].

#### 3.1.2. Development of Orally Available Opioidmimetic Analgesics by Dimerization of Dmt with Diaminoalkylpyrazinones

**Figure 2:**
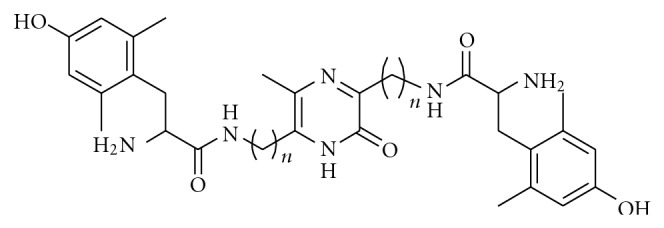
Structure of 3,6-*bis *[Dmt-NH-(CH_2_)_n_]-5-methyl-2(*1H*)-pyrazinones.

The inability of opioid peptides to be transported through epithelial membranes in the gastrointestinal tract and pass the blood-brain barrier limits their effectiveness for oral application in an antinociceptive treatment regime. To overcome this limitation, we enhanced the hydrophobicity and maintained the aqueous solubility properties of ligands by employing two identical N-termini. This consisted of Dmt coupled to a pyrazinone ring platform by means of alkyl chains to yield the class of 3,6-*bis*-[Dmt-NH-(CH_2_)_n_]-5-methyl-2(1*H*)-pyrazinones (Figure 2) [19]. Their receptor affinities and *in vitro* bioactivities are summarized in Tables 2 and 4, respectively. The 3,6-*bis*-[Dmt-NH-(CH_2_)_n_]-5-methyl-2(1*H*)-pyrazinone compounds exhibited high affinity to both *μ*- (**56–58**: *K_i_μ* = 0.04–0.12 nM) and *δ*-opioid receptors (**55–58**: *K_i_δ* = 7.3–23.2 nM). Compound** 57**, 3,6-*bis*-[Dmt-NH-(CH_2_)_3_]-5-methyl-2(1*H*)-pyrazinone exhibited the highest affinity (*K_i_μ* = 0.042 nM) that was ca. 3-fold greater than that of either 3,6-*bis*-[Dmt-NH-(CH_2_)_2_]-5-methyl-2(1*H*)-pyrazinone (**56**) or 3,6-*bis*-[Dmt-NH-(CH_2_)_4_]-5-methyl-2(1*H*)-pyrazinone (**58**) and nearly 30 times greater than that of 3,6-*bis*-[Dmt-NH-CH_2_]-5-methyl-2(1*H*)-pyrazinone (**55**). Thus, the length of the interposing alkyl chain determines the efficacy of receptor binding: propyl > ethyl, butyl ≫ methyl. Compounds **55**–**58 **were biologically active and generally reflected the values obtained for the affinity constants: **57 **was the most active (GPI, IC_50_ = 1.33 nM) and more potent than the *bis-*[Dmt-NH]-alkyl compounds (**51–54**: GPI IC_50_ = 3.08–2,844 nM) [18] and was a *μ*-selective agonist without measurable *δ*-bioactivity. Compound **58**, which exhibited 30% less *μ*-agonism than **57**, had weak *δ*-agonism (MVD, IC_50_ = 41.5 nM). Similar to the *bis*-[Dmt-NH]-alkyl compounds (**51**–**54**: pA_2_ = 5.5–6.5) [18], compounds **55 **and** 56 **were weak *δ*-antagonists (pA_2_ = 6.47 and 6.56, resp.).

Compound **57** produced naloxone reversible analgesia by i.c.v., s.c. and oral (po) administration. While i.c.v. analgesia was 50- and 20-fold more potent than morphine in the tail-flick and hot-plate tests, respectively, both s.c. and p.o. were somewhat less active than morphine. These results demonstrated that compound **57** crossed epithelial membrane barriers in both the intestine and microcapillaries in mouse brain to interact with brain *μ*-opioid receptors. Similar conclusions were obtained by Igarashi et al. [31] and Koda et al. [32]. These results indicated that pyrazinone derivatives could be potential candidates for clinical and therapeutic applications in the treatment of pain arising from postoperative procedure or cancer, associated with birth, or act as possible veterinary drugs.

### 3.2. Development of *μ*- and *δ*-Opioid Receptor Antagonists by Dimerization of Dmt-Tic with Diaminoalkanes or Diaminoalkylpyrazinones

We expanded our studies with Dmt through the synthesis and analysis of the biological properties of unique series of dimeric H-Dmt-Tic (2′,6′-dimethyl-l-tyrosyl-1,2,3,4-tetrahydroisoquinoline-3-carboxylic acid) analogues linked either through diaminoalkanes of variable length (**66**–**68**) or by symmetric or asymmetric 3,6-diaminoalkyl-5-methyl-2(1*H*)-pyrazinone derivatives (**59–65**). Salvadori et al. [14] first reported that H-Dmt-Tic-OH had not only *δ* high affinity (*K_i_δ* = 0.022 nM) but also extraordinary selectivity for the *δ*-opioid receptor (*K_i_μ*/*K_i_δ* = 150,800) without interaction to *κ*-opioid receptors and exhibited *δ*-selective antagonism.

As summarized in Table 2, most of the compounds exhibited high subnanomolar affinities to *δ*-opioid receptors (*K_i_δ* = 0.095–0.323 nM) independent of the spacer used, except compound **68** (*K_i_δ* = 1.53 nM); *μ*-receptor affinities fell within the low nanomolar range of 1–5 nM [20]. Compared to H-Dmt-Tic-OH [14], our observed *μ*-affinities increased by several orders of magnitude.

In the series of dimeric H-Dmt-Tic-OH, ligands (**59**–**70**) listed in Table 4 were devoid of *δ*-opioid receptor mediated agonism; all the compounds were exceptionally potent *δ*-antagonists with pA_2_ values ranging from 10.42 to 11.28, which represent orders of magnitude greater than that of both naltrindole (pA_2_ = 9.20) and H-Dmt-Tic-OH (pA_2_ = 8.48). In contrast to their *μ*-opioid receptor affinities (Table 2), the compounds exhibited very weak to nonexistent *μ*-agonism, especially, **69 **and **70**, which exhibited pure and potent *δ*- and *μ*-antagonism in the same molecule. In fact, the *μ*-opioid receptor antagonism of **69** and **70 **exceeds that of other known peptidic [33] and nonpeptidic [34] antagonists.

The extraordinary dual *δ*/*μ*-antagonism of **69** and **70** qualifies these compounds as potential pharmacological tools for application in the clinical and therapeutic treatment of drug addiction and alcohol dependency. Considering that the *bis*-Dmt analogues containing alkylpyrazinone are orally active and pass through the blood-brain barrier [19, 31, 32], we would anticipate that **69** and **70** might show similar properties or may be even more potent due to their increased hydrophobicity [35].

## 4. Conclusion

Based on the structure of endomorphins (H-Tyr-Pro-Trp/Phe-Phe-NH_2_), which exhibited very high selectivity toward *μ*-opioid receptors, we developed various analogues and examined their activities by alterations of a specific residue. From the studies on the stereoisomers of EM-2, [D-Pro^2^]EM-2 (**12**) exhibited more potent and prolonged analgesia [7] although it exhibited low *μ*-affinity [5], indicating an enhanced bioactivity due to a presumed resistance to enzymatic degradation by dipeptidyl peptidase IV [8]. Substitution of Tyr^1^ by Dmt yielded [Dmt^1^]EM-1 (**19**) and [Dmt^1^]EM-2 (**20**): the former, containing Trp^3^, had mixed *μ*-agonism/*δ*-antagonism properties, and the latter, with Phe^3^, exhibited dual *μ*/*δ*-agonism. The differences between bulkiness of Trp and Phe defined their biofunctional properties, suggesting the existence of fine differences in the stereo geometry of the ligand-binding site between *μ*- and *δ*-opioid receptors. These data provided us with methodology to design ligands with agonism or antagonism towards their respective receptors. Thus, we could develop various compounds with dual *μ*-/*δ*-agonism or mixed *μ*-agonism/*δ*-antagonism in the same molecule.

On the other hand, alkylation of the N-termini of [Dmt^1^]EM-1 and [Dmt^1^]EM-2 converted *μ*-agonists into neutral acting *μ*-antagonists: [*N*-allyl-Dmt^1^]EM-1 (**47**) and [*N*-allyl-Dmt^1^]EM-2 (**48**) exhibited potent and highly selective *μ*-antagonism without inverse agonism, suggesting a potential clinical application in the treatment of drug addiction and alcohol abuse without adverse effects [10, 26, 27, 35]. Similarly ligands with two Dmt residues separated by diaminoalkane or diaminoalkylpyrazinone produced orally available opioidmimetic analgesics. The compound 3,6-*bis*-[Dmt-NH-(CH_2_)_3_]-5-methyl-2(1*H*)-pyrazinone (**57**), *μ*-selective agonist, produced naloxone reversible analgesia following oral administration, with a potency that was 42% and 24% compared to morphine in tail-flick and hot-plate tests in mice, respectively. These results demonstrated that **57** passed through membranes in the gastrointestinal tract and the blood-brain barrier [19]. This observation paves the way for its clinical and therapeutic application in the treatment of pain. Dimerization of potent and *δ*-selective antagonist H-Dmt-Tic-OH [14] separated by diaminoalkane or 3,6-diaminoalkylpyrazinone produced the dual *μ*/*δ*-antagonists, *bis*-[*N,N*-dimethyl-Dmt-Tic-NH]hexane (**69**) and 3,6-*bis*-[*N,N*-dimethyl-Dmt-Tic-NH-(CH_2_)_3_]-5-methyl-2(1*H*)-pyrazinone (**70**) [20]. These extraordinary dual *μ*/*δ*-antagonists (**69** and **70**) also qualify as potential drugs with clinical and therapeutic applications.

## Figures and Tables

**Table 1:** Opioid receptor affinities of opioid ligands **1–50**.

Nos.	Compounds		*K_i_μ* (nM)	*K_i_δ* (nM)	*K_i_δ*/*K_i_μ*	Reference
**1**	H-Tyr-Pro-Trp-Phe-NH_2_	(EM-1)	0.36	1,510	4,190	[4]
**2**	H-Tyr-Pro-Phe-Phe-NH_2_	(EM-2)	0.69	9,230	13,380	[4]
**3**	d-d-d-d	(EM-2)	1,041	16,579	98	[5]
**4**	l-l-d-d	(EM-2)	24.3	1,249	51	[5]
**5**	d-d-l-l	(EM-2)	2,755	19,459	7	[5]
**6**	d-l-l-l	(EM-2)	32.1	4,121	128	[5]
**7**	l-d-d-d	(EM-2)	2,013	13,278	6.6	[5]
**8**	l-l-l-d	(EM-2)	45.9	8,159	177	[5]
**9**	d-d-d-l	(EM-2)	107.9	7,203	67	[5]
**10**	l-l-d-l	(EM-2)	203.2	4,230	21	[5]
**11**	d-d-l-d	(EM-2)	7,051	18,624	2.6	[5]
**12**	l-d-l-l	(EM-2)	512.4	30,641	60	[5]
**13**	d-l-d-d	(EM-2)	363.5	21,264	58	[5]
**14**	d-l-l-d	(EM-2)	557.3	4,187	7.5	[5]
**15**	l-d-d-l	(EM-2)	4,707	16,662	3.5	[5]
**16**	d-l-d-l	(EM-2)	651.6	14,584	22	[5]
**17**	l-d-l-d	(EM-2)	1,311	26,211	20	[5]
**18**	H-Tyr-Pro-Phe-NH_2_		46.1	15,900	344	[9]
**19**	H-Dmt-Pro-Trp-Phe-NH_2_		0.054	5.6	104	[10]
**20**	H-Dmt-Pro-Phe-Phe-NH_2_		0.15	28.2	188	[9]
**21**	H-Dmt-Pro-Phe-NH_2_		0.12	53.2	443	[9]
**22**	H-Dmt-Pro-Phe-NH-C_2_H_4_-Ph		0.51	18	35	[9]
**23**	H-Dmt-Pro-Phe-NH-Bzl		0.52	13.8	27	[11]
**24**	H-Dmt-Pro-Phe-NH-Ph		1.11	20.6	19	[11]
**25**	H-Dmt-Pro-Phe-NH-4-Pyr		0.36	52.6	146	[11]
**26**	H-Dmt-Pro-Phe-NH-3-Pyr		0.17	287	1,690	[11]
**27**	H-Dmt-Pro-Phe-NH-2-Pyr		0.13	157	1,210	[11]
**28**	H-Dmt-Pro-Phe-NH-1-Nph		0.29	19.9	68	[11]
**29**	H-Dmt-Pro-Phe-NH-3-Qln		0.33	190	575	[11]
**30**	H-Dmt-Pro-Phe-NH-5-Qln		0.11	30	272	[11]
**31**	H-Dmt-Pro-Phe-NH-6-Qln		0.22	46.6	212	[11]
**32**	H-Dmt-Pro-Phe-NH-8-Qln		0.49	33.1	68	[11]
**33**	H-Dmt-Pro-Phe-NH-5-Isq		0.19	98.3	517	[11]
**34**	H-Mmt-Pro-Phe-Phe-NH_2_		0.132	528.6	4,005	[12]
**35**	H-Emt-Pro-Phe-Phe-NH_2_		0.063	55.7	884	[12]
**36**	H-Imt-Pro-Phe-Phe-NH_2_		0.15	190	1,226	[12]
**37**	H-Det-Pro-Phe-Phe-NH_2_		0.084	69.7	830	[12]
**38**	H-Dit-Pro-Phe-Phe-NH_2_		2.29	105	46	[12]
**39**	H-Tmt-Pro-Phe-Phe-NH_2_		0.111	593.5	5,347	[12]
**40**	H-Dmt-Pro-Mmp-Phe-NH_2_		0.18	4.61	26	[13]
**41**	H-Dmt-Pro-^3,5^Dmp-Phe-NH_2_		0.11	11.6	105	[13]
**42**	H-Dmt-Pro-Dmp-Phe-NH_2_		0.069	2.27	33	[13]
**43**	H-Dmt-Pro-Dmt-Phe-NH_2_		0.092	80.8	878	[13]
**44**	H-Dmt-Pro-Tmp-Phe-NH_2_		0.18	1.83	10	[13]
**45**	H-Dmt-Pro-Emp-Phe-NH_2_		0.21	3.03	14	[13]
**46**	H-Dmt-Pro-Imp-Phe-NH_2_		0.32	4.61	14	[13]
**47**	[*N*-allyl-Dmt^1^]EM-1		0.26	10.3	40	[10]
**48**	[*N*-allyl-Dmt^1^]EM-2		0.45	560	1,244	[10]
**49**	1,6-*bis*[*N*-allyl-Dmt-NH]hexane		12.4	51.5	4	[10]
**50**	3,6-*bis*[*N*-allyl-Dmt-NH-propyl]-5-methyl-2(*1H*)-pyrazinone		6.94	77.8	11	[10]

Opioid receptor affinities are determined using rat brain P_2_  synaptosomal preparations with [^3^H]DAMGO for *μ*-opioid receptors and [^3^H]DPDPE for  *δ*-opioid receptors.

**Table 2:** Opioid receptor affinities of opioid ligands **51–70**.

Nos.	Compounds	*K_i_μ*(nM)	*K_i_δ*(nM)	*K_i_δ*/*K_i_μ*	Reference
**51**	Dmt-NH-(CH_2_)_2_-NH-Dmt	1.43	115.7	81	[18]
**52**	Dmt-NH-(CH_2_)_4_-NH-Dmt	0.041	53.4	1302	[18]
**53**	Dmt-NH-(CH_2_)_6_-NH-Dmt	0.053	46.1	870	[18]
**54**	Dmt-NH-(CH_2_)_8_-NH-Dmt	0.19	14.8	78	[18]
**55**	3,6-*bis*[Dmt-NH-CH_2_]-5-methyl-2(*1H*)-pyrazinone	1.16	15.7	13.5	[19]
**56**	3,6-*bis*[Dmt-NH(CH_2_)_2_]-5-methyl-2(*1H*)-pyrazinone	0.115	7.26	63	[19]
**57**	3,6-*bis*[Dmt-NH(CH_2_)_3_]-5-methyl-2(*1H*)-pyrazinone	0.042	13.2	307	[19]
**58**	3,6-*bis*[Dmt-NH(CH_2_)_4_]-5-methyl-2(*1H*)-pyrazinone	0.114	23.2	204	[19]
**59**	3,6-*bis*[Dmt-Tic-NH-CH_2_]-5-methyl-2(*1H*)-pyrazinone	3.76	0.163	0.043	[20]
**60**	3,6-*bis*[Dmt-Tic-NH(CH_2_)_2_]-5-methyl-2(*1H*)-pyrazinone	2.83	0.095	0.034	[20]
**61**	3,6-*bis*[Dmt-Tic-NH(CH_2_)_3_]-5-methyl-2(*1H*)-pyrazinone	3.08	0.155	0.05	[20]
**62**	3,6-*bis*[Dmt-Tic-NH(CH_2_)_4_]-5-methyl-2(*1H*)-pyrazinone	1.74	0.323	0.185	[20]
**63**	3-[Dmt-Tic-NH(CH_2_)_3_]-6-[Dmt-Tic-NH(CH_2_)_4_]-5-methyl-2(*1H*)-pyrazinone	1.56	0.16	0.1	[20]
**64**	3-[Dmt-Tic-NH(CH_2_)_4_]-6-[Dmt-Tic-NH(CH_2_)_3_]-5-methyl-2(*1H*)-pyrazinone	2.28	0.092	0.04	[20]
**65**	3-[Dmt-Tic-NH(CH_2_)_2_]-6-[Dmt-Tic-NH(CH_2_)_4_]-5-methyl-2(*1H*)-pyrazinone	1.37	0.107	0.078	[20]
**66**	*bis*[Dmt-Tic-NH]butane	5.72	0.124	0.021	[20]
**67**	*bis*[Dmt-Tic-NH]hexane	1.79	0.129	0.072	[20]
**68**	*bis*[Dmt-Tic-NH]decane	4.86	1.53	0.315	[20]
**69**	*bis*[*N,N*-dimethyl-Dmt-Tic-NH]	2.21	0.06	0.027	[20]
	hexane				
**70**	3,6-*bis*[*N,N*-dimethyl-Dmt-Tic-NH-	1.68	0.287	0.17	[20]
	propyl]-5-methyl-2(*1H*)-pyrazinone				

Opioid receptor affinities are determined using rat brain P_2_  synaptosomal preparations with [^3^H]DAMGO for *μ*-opioid receptors and [^3^H]DPDPE for  *δ*-opioid receptors.

**Table 3:** Functional bioactivities of opioid ligands **1, 2,** and **19–50**.

			GPI		MVD		

Nos.	Compounds		IC_50_ (nM)^a^	pA_2_^b^	IC_50_ (nM)	pA_2_	Reference

**1**	H-Tyr-Pro-Trp-Phe-NH_2_	(EM-1)	4.03	—^c^	283	—	[10]
**2**	H-Tyr-Pro-Phe-Phe-NH_2_	(EM-2)	6.88	—	344	—	[10]
**19**	H-Dmt-Pro-Trp-Phe-NH_2_		0.27	—	>10,000	8.6	[10]
**20**	H-Dmt-Pro-Phe-Phe-NH_2_		0.07	—	1.87	—	[9]
**21**	H-Dmt-Pro-Phe-NH_2_		2.33	—	113	—	[9]
**22**	H-Dmt-Pro-Phe-NH-C_2_H_4_-Ph		5.03	—	>10,000	7.05	[9]
**23**	H-Dmt-Pro-Phe-NH-Bzl		22	—	>10,000	7.18	[11]
**24**	H-Dmt-Pro-Phe-NH-Ph		37.7	—	>10,000	6.94	[11]
**25**	H-Dmt-Pro-Phe-NH-4-Pyr		11.8	—	>10,000	6.52	[11]
**26**	H-Dmt-Pro-Phe-NH-3-Pyr		72.8	—	>10,000	6.33	[11]
**27**	H-Dmt-Pro-Phe-NH-2-Pyr		15	—	>10,000	6.7	[11]
**28**	H-Dmt-Pro-Phe-NH-1-Nph		0.49	—	5.47	—	[11]
**29**	H-Dmt-Pro-Phe-NH-3-Qln		9.14	—	>10,000	5.93	[11]
**30**	H-Dmt-Pro-Phe-NH-5-Qln		0.26	—	0.616	5.88	[11]
**31**	H-Dmt-Pro-Phe-NH-6-Qln		6.21	—	>10,000	5.41	[11]
**32**	H-Dmt-Pro-Phe-NH-8-Qln		445	—	2,981	6.14	[11]
**33**	H-Dmt-Pro-Phe-NH-5-Isq		0.94	—	>10,000	6.12	[11]
**34**	H-Mmt-Pro-Phe-Phe-NH_2_		0.924	—	28.7	++^d^	[12]
**35**	H-Emt-Pro-Phe-Phe-NH_2_		0.623	—	1.08	+^e^	[12]
**36**	H-Imt-Pro-Phe-Phe-NH_2_		10.6	—	601	+	[12]
**37**	H-Det-Pro-Phe-Phe-NH_2_		0.903	—	47.1	+	[12]
**38**	H-Dit-Pro-Phe-Phe-NH_2_		299	—	>10,000	ND^f^	[12]
**39**	H-Tmt-Pro-Phe-Phe-NH_2_		2.31	—	46.4	++	[12]
**40**	H-Dmt-Pro-Mmp-Phe-NH_2_		0.16	—	>10,000	6.59	[13]
**41**	H-Dmt-Pro-^3,5^Dmp-Phe-NH_2_		14.4	—	>10,000	6.77	[13]
**42**	H-Dmt-Pro-Dmp-Phe-NH_2_		0.12	—	>10,000	8.15	[13]
**43**	H-Dmt-Pro-Dmt-Phe-NH_2_		1.94	—	>10,000	7.06	[13]
**44**	H-Dmt-Pro-Tmp-Phe-NH_2_		0.21	—	>10,000	9.05	[13]
**45**	H-Dmt-Pro-Emp-Phe-NH_2_		0.17	—	0.51	—	[13]
**46**	H-Dmt-Pro-Imp-Phe-NH_2_		0.2	—	5.56	—	[13]
**47**	[*N*-allyl-Dmt^1^]EM-1		>10,000	8.18	>10,000	7.32	[10]
**48**	[*N*-allyl-Dmt^1^]EM-2		>10,000	8.59	>10,000	6.32	[10]
**49**	1,6-*bis*[*N*-allyl-Dmt-NH]hexane		>10,000	7.23	>10,000	6.83	[10]
**50**	3,6-*bis*[*N*-allyl-Dmt-NH-propyl]-5-methyl-2(*1H*)-pyrazinone		>10,000	7.17	>10,000	6.38	[10]

^a^IC_50_ value is the concentration required to 50% inhibition of the electrically induced contraction in a muscle. ^b^pA_2_ is the negative log of the molar concentration required to double the agonist IC_50_ value in order to achieve the original response. ^c^Not tested. ^d,e^Antagonism by CTAP (200 nM) with the percent recovery of electrically evoked contraction: ++, >50%; +, <50%. ^f^Not detected.

**Table 4:** Functional bioactivities of opioid ligands **51–70**.

		GPI		MVD		

Nos.	Compounds	IC_50_ (nM)^a^	pA_2_^b^	IC_50_ (nM)	pA_2_	Reference

**51**	Dmt-NH-(CH_2_)_2_-NH-Dmt	2,844	—^c^	>10,000	5.5	[18]
**52**	Dmt-NH-(CH_2_)_4_-NH-Dmt	5.33	—	>10,000	5.8	[18]
**53**	Dmt-NH-(CH_2_)_6_-NH-Dmt	3.08	—	>10,000	6.1	[18]
**54**	Dmt-NH-(CH_2_)_8_-NH-Dmt	53.7	—	>10,000	6.4	[18]
**55**	3,6-*bis*[Dmt-NH-CH_2_]-5-methyl-2(*1H*)-pyrazinone	1,695	—	>10,000	6.47	[19]
**56**	3,6-*bis*[Dmt-NH(CH_2_)_2_]-5-methyl-2(*1H*)-pyrazinone	12.9	—	>10,000	6.56	[19]
**57**	3,6-*bis*[Dmt-NH(CH_2_)_3_]-5-methyl-2(*1H*)-pyrazinone	1.33	—	>10,000	ND^d^	[19]
**58**	3,6-*bis*[Dmt-NH(CH_2_)_4_]-5-methyl-2(*1H*)-pyrazinone	1.9	—	41.5	ND	[19]
**59**	3,6-*bis*[Dmt-Tic-NH-CH_2_]-5-methyl-2(*1H*)-pyrazinone	>10,000	ND	>10,000	11.22	[20]
**60**	3,6-*bis*[Dmt-Tic-NH(CH_2_)_2_]-5-methyl-2(*1H*)-pyrazinone	>10,000	6.78	>10,000	10.73	[20]
**61**	3,6-*bis*[Dmt-Tic-NH(CH_2_)_3_]-5-methyl-2(*1H*)-pyrazinone	7,025	ND	>10,000	10.56	[20]
**62**	3,6-*bis*[Dmt-Tic-NH(CH_2_)_4_]-5-methyl-2(*1H*)-pyrazinone	>10,000	ND	>10,000	11.06	[20]
**63**	3-[Dmt-Tic-NH(CH_2_)_3_]-6-[Dmt-Tic-NH(CH_2_)_4_]-5-methyl-2(*1H*)-pyrazinone	>10,000	ND	>10,000	10.6	[20]
**64**	3-[Dmt-Tic-NH(CH_2_)_4_]-6-[Dmt-Tic-NH(CH_2_)_3_]-5-methyl-2(*1H*)-pyrazinone	>10,000	6.95	>10,000	10.47	[20]
**65**	3-[Dmt-Tic-NH(CH_2_)_2_]-6-[Dmt-Tic-NH(CH_2_)_4_]-5-methyl-2(*1H*)-pyrazinone	>10,000	ND	>10,000	10.99	[20]
**66**	*bis*[Dmt-Tic-NH]butane	>10,000	6.99	>10,000	10.51	[20]
**67**	*bis*[Dmt-Tic-NH]hexane	2,715	ND	>10,000	10.62	[20]
**68**	*bis*[Dmt-Tic-NH]decane	5,425	ND	>10,000	10.97	[20]
**69**	*bis*[*N,N*-dimethyl-Dmt-Tic-NH]hexane	>10,000	8.34	>10,000	11.28	[20]
**70**	3,6-*bis*[*N,N*-dimethyl-Dmt-Tic-NH-propyl]-5-methyl-2(*1H*)-pyrazinone	>10,000	7.71	>10,000	10.42	[20]

^a^IC_50_ value is the concentration required to 50% inhibition of the electrically induced contraction in a muscle. ^b^pA_2_ is the negative log of the molar concentration required to double the agonist IC_50_ value in order to achieve the original response. ^c^Not tested. ^d^Not determined.

## Abbreviations

### Abbreviations

Bu*^t^*: *tert*-Butyl
HBTU: *O*-Benzotriazole-1-yl-*N*,*N*,*N*′, *N*′-tetramethyluronium hexafluorophosphate
DIPEA: Diisopropylethylamine
HOBt: 1,2,3-Benzotriazole-1-ol
NMM: *N*-Methylmorpholine
NMP: *N*-Methylpyrrolidone
TFA: Trifluoroacetic acid
Ph: Phenyl
Bzl: Benzyl
4-Py: 4-Pyridyl
3-Py: 3-Pyridyl
2-Py: 2-Pyridyl
1-Nph: 1-Naphthyl
3-Qln: 3-Quinolyl
5-Qln: 5-Quinolyl
6-Qln: 6-Quinolyl
8-Qln: 8-Quinolyl
5-Isq: 5-Isoquinolyl
Dmt: 2′,6′-Dimethyl-l-tyrosine
Emt: 2′-Ethyl-6′-methyl-l-tyrosine
Mmt: 2′-Methyl-l-tyrosine
Imt: 2′-Isopropyl-6′-methyl-l-tyrosine
Det: 2′,6′-Diethyl-l-tyrosine
Dit: 2′,6′-Diisopropyl-l-tyrosine
Tmt: 2′,3′,6′-Trimethyl-l-tyrosine
Dmp: 2′,6′-Dimethyl-l-phenylalanine
Mmp: 2′-Methyl-l-tyrosine
^3,5^Dmp: 3′,5′-Dimethyl-l-phenylalanine
Tmp: 2′,4′,6′-Trimethyl-l-phenylalanine
Emp: 2′-Ethyl-6′- methyl-l-phenylalanine
Imp: 2′-Isopropyl-6′-methyl-l-phenylalanine
IPSC: Inhibitory postsynaptic currents.
